# Immunoregulatory extracellular vesicles in multiple myeloma patients’ peripheral blood

**DOI:** 10.20517/evcna.2025.180

**Published:** 2026-07-06

**Authors:** Irma Airoldi, Debora Soncini, Danilo Marimpietri, Fabio Malavasi, Alberto L. Horenstein, Matteo Garibotto, Michele Cea, Fabio Morandi

**Affiliations:** ^1^UOSD Laboratory of Cell Therapies, IRCCS Istituto Giannina Gaslini, Genova 16147, Italy.; ^2^Clinic of Hematology, Department of Internal Medicine (DiMI), University of Genoa, Genova 16132, Italy.; ^3^IRCCS Ospedale Policlinico San Martino, Genova 16132, Italy.; ^4^Lab of Immunogenetics, Department of Medical Sciences, University of Torino, Torino 10126, Italy.; ^5^Fondazione Ricerca Molinette ETS, Torino 10126, Italy.; ^#^These authors contributed equally to this work as first authors.; ^§^These authors contributed equally to this work as senior authors.

**Keywords:** Multiple myeloma, extracellular vesicles, adenosinergic ectoenzymes

## Abstract

**Aim:** Extracellular vesicles (EVs) mediate cell-to-cell communication and co-contribute in cancer progression and modulation of immune responses. The aim of this work was to characterize the phenotype and function of EVs isolated from the peripheral blood (PB) of multiple myeloma (MM) patients, with particular attention to adenosinergic ectoenzymes.

**Methods:** EVs were isolated by centrifugation of PB plasma samples from newly diagnosed (ND) MM patients and healthy donors (HD). EVs were characterized by nanoparticle tracking analysis (NTA). Flow cytometry was used to assess the expression of CD138 (a tumor-associated antigen), nicotinamide phosphoribosyltransferase/pre-B cell colony enhancing factor (NAMPT/PBEF1), and adenosinergic ectoenzymes CD38, CD39, CD203a, and CD73. Functional studies were performed by assessing T-cell proliferation *in vitro* using carboxyfluorescein succinimidyl ester (CFSE) dilution and flow cytometry, with or without EVs to the culture.

**Results:** The PB EVs size was comparable between MM patients and HDs, whereas EV concentration was significantly higher in MM patients. EVs from MM patients expressed higher levels of CD138, PBEF, CD38, and CD73 than those from HDs. In addition, CD138^+^ EVs from MM patients expressed higher levels of PBEF, CD38, CD39, and CD73 than CD138^-^ counterparts. Finally, MM-derived EVs inhibited T-cell proliferation *in vitro*. This inhibition correlated with the expression of CD138 and CD203a.

**Conclusion:** This study provides the first phenotypic and functional characterization of EVs obtained from the PB of MM patients. The presence of functional adenosinergic molecules may represent a novel immune-escape mechanism operating at distant sites. Nonetheless, further studies are required to define specific contributions of adenosinergic ectoenzymes and other immune-regulatory mechanisms.

## INTRODUCTION

The clonal expansion of plasma cells (PCs) in the bone marrow (BM) is the hallmark of multiple myeloma (MM), a hematological tumor that typically affects subjects over 60 years of age and represents about 0.9% of all cancers worldwide, contributing to about 1% of cancer-related deaths^[1]^. Although the etiology of MM has not been fully elucidated, multiple cytogenetic alterations, such as chromosomal deletions and/or translocations, are present and correlated with disease progression and high-risk MM^[2]^. Asymptomatic forms like monoclonal gammopathy of undetermined significance (MGUS) and smoldering multiple myeloma (SMM) represent premalignant stages that may advance to symptomatic MM. Disease progression is characterized by severe and irreversible impairment of vital organs, including hypercalcemia, renal failure, anemia, and lesions in the bones^[2]^.

The expression and function of adenosinergic pathways in MM have been previously described by our group and others^[3,4]^. The canonical pathway for adenosine (ADO) production is initiated by the nucleoside triphosphate diphosphohydrolase/CD39, which converts adenosine triphosphate (ATP) into adenosine monophosphate (AMP)^[5]^. Subsequently, the hydrolysis of AMP to ADO is mediated by ecto-5’-nucleotidase/CD73^[6,7]^. In contrast, the non-canonical pathway, whose efficiency increases at low pH, starts with the conversion of NAD^+^ to adenosine diphosphate ribose (ADPR) mediated by the nicotinamide adenine dinucleotide (NAD^+^)-glycohydrolase/CD38. ADPR is subsequently converted to AMP by CD203a/ENPP1 (PC-1), which has been characterized in both tumor and immune cells^[8]^, although the conversion of ADPR to AMP was first demonstrated functionally by our group^[9]^. AMP is finally metabolized to ADO by CD73^[7,10,11]^, which catalyzes the final step of both adenosinergic pathways. Functionally, adenosinergic ectoenzymes are key players in the establishment of an immune-suppressive BM microenvironment through a local production of ADO^[4,12]^. These processes are facilitated by nicotinamide phosphoribosyltransferase/pre-B cell colony enhancing factor (NAMPT/PBEF1), a key enzyme involved in the conversion of nicotinamide and 5-phosphoribosyl-1-pyrophosphate into nicotinamide mononucleotide, which is subsequently transformed into NAD^+^. This pathway supports not only glycolysis but also the activity of NAD^+^-dependent enzymes and DNA repair mechanisms^[13-16]^. NAMPT/PBEF1 expression is increased in both solid and hematological tumors^[17,18]^ as well as in inflammatory diseases^[19,20]^.

We have recently reported that many of these molecules are also expressed on extracellular vesicles (EVs) obtained from BM plasma samples of MM patients^[21]^. Several studies have described these EVs as resembling the phenotypic and functional characteristics of their cells of origin. Furthermore, their role in multiple processes related to MM pathogenesis, including modulation of immune responses, progression of the disease, therapy resistance, osteolysis, angiogenesis, and formation of the metastatic niche has been addressed^[22-25]^. However, to date, no information is available on tumor-derived EVs present in the peripheral blood (PB) of MM patients. Therefore, the aim of this study was to characterize these EVs in terms of size, number, and phenotype, and to assess whether they exert immune-regulatory functions *in vitro*. The data obtained in this study indicate that tumor-derived EVs can be also detected in the PB of newly diagnosed MM (NDMM) patients and may contribute to tumor-associated immune suppression.

## METHODS

### Patient characteristics and enrollment criteria

PB samples were collected from 13 NDMM patients enrolled at Istituto di Ricerca e Cura a Carattere Scientifico (IRCCS) Azienda Ospedaliera Metropolitana (AOM), Genova, Italy. Patients were eligible for enrollment if they met the following inclusion criteria: age ≥ 50 years; confirmed diagnosis of MM according to International Myeloma Working Group (IMWG) diagnostic criteria; PB samples were collected at the time of clinical diagnosis and prior to the initiation of any anti-MM treatment, processed for plasma isolation, and subsequently cryopreserved as plasma samples. Exclusion criteria included the presence of active acute infections at the time of blood collection, as well as known autoimmune or overt inflammatory diseases, in order to minimize confounding effects on EV profiles and immune functional assays.

Healthy donors’ (HD) PB samples (*n* = 12) were used as controls. All samples were collected after written informed consent was obtained from all subjects, in accordance with the Declaration of Helsinki. The study was approved by the Ethics Committee (CER Liguria: 409/2021 - DB ID 11602). The clinical features of MM patients are reported in Table 1.

**Table 1:** Clinical characteristics of MM patients

**Age at diagnosis**	**Myeloma subtype**	**R-ISS-stage**	**CA**
71	IgG lambda	II	None
77	IgA kappa	II	del(13q)
71	IgG kappa	II	del(13q)
81	IgA kappa	II	N/A
78	IgA kappa	II	del(13q), (1q) gain
66	IgG kappa	III	5p15-9q22-15CEN hyperdiploid status
87	IgG kappa	II	N/A
82	IgG lambda	I-II	N/A
62	IgG kappa	II	N/A
60	IgA lambda	II	(1q) gain
69	IgA lambda + IgG kappa	III	N/A
68	IgG kappa	N/A	N/A
80	IgG kappa	I	None

CA: Chromosomal abnormalities; N/A: not available; R-ISS: Revised International Staging System.

### Isolation and characterization of EVs

Blood samples were centrifuged at 3,000 × *g* for 15 min at 4 °C to obtain PB plasma, which was divided into aliquots (300 μL each) and cryopreserved at -80 °C. Isolation of EVs from thawed samples was performed as previously reported^[4]^. For some patients, multiple EV preparations were obtained from different plasma aliquots, resulting in a total of 22 EV preparations analyzed. Briefly, PB plasma samples were diluted 1:3 with phosphate buffered saline (PBS), and platelets and debris were removed by centrifugation at 3,000 × *g* for 15 min at 4 °C. The collected supernatants were analyzed to determine EV size and concentration with the employment of a NanoSight NS500 instrument [nanoparticle tracking analysis (NTA) 2.3 software, 488 nm laser]. Finally, EVs were enriched by centrifugation (20,000 × *g* at 4 °C for 1 h). After washing with PBS, EVs were resuspended in Magnetic-Activated Cell Sorting (MACS) buffer (PBS/EDTA with 0.5% BSA, 50 μL). For functional experiments, enriched EVs were resuspended in sterile serum-free RPMI-1640 medium.

### Flow cytometric analysis

The number of EVs enriched from 300 μL of PB plasma samples, and then subjected to flow cytometric analysis, varied between subjects. CD138 and NAMPT/PBEF1 expression was evaluated using rat Alexa Fluor 488-conjugated anti-CD138 mAb (#359103) and rat Alexa Fluor 647-conjugated anti-PBEF1 mAb (#362616), respectively. The expression of ectoenzymes was evaluated using anti-CD38 (#IB4) and anti-CD73 (#CB73) monoclonal antibodies (mAbs), generated in our laboratory and conjugated with fluorescein isothiocyanate (FITC), phycoerythrin (PE), or allophycocyanin (APC) fluorochromes by Aczon (Bologna, Italy). Anti-CD203a (PC-1, clone #3E8) was kindly provided by J. Goding, and anti-CD39 PE-Cy7 mAbs were purchased from eBiosciences. Irrelevant isotype- and fluorochrome-matched mAbs were obtained from Beckman Coulter.

EVs were incubated with mAbs (20 min at 4 °C in the dark), washed, centrifuged (20,000 × *g* at 4 °C for 1 h), and resuspended in MACS buffer (200 μL). A Gallios flow cytometer (Beckman Coulter) was used for data acquisition, and Kaluza software version 2.1 (Beckman Coulter) was used for data analysis. At least 2,000 events per sample were acquired using a forward- and side-scatter-based gating strategy to exclude debris background. The expression of ectoenzymes and NAMPT/PBEF1 in EVs specifically derived from MM cells was investigated by gating on CD138^+^ EVs. Results were expressed as mean relative fluorescence intensity (MRFI), calculated as the ratio between the mean fluorescence intensity obtained with the specific antibody and that obtained with the corresponding irrelevant isotype control.

### T-cell proliferation assay

Mononuclear cells (MNCs) were isolated from buffy coat preparations obtained from healthy donors (HDs; *n* = 3) enrolled at AOM, following written informed consent. Isolation was performed using Ficoll-Paque density gradient centrifugation (800 × *g* for 30 min at room temperature). The CellTrace^TM^ carboxyfluorescein succinimidyl ester (CFSE) Cell Proliferation Kit (Thermo Fisher Scientific) was used to assess T-cell proliferation. Briefly, MNCs were stained with CFSE (2 μM) for 15 min at 37 °C, washed, and resuspended in RPMI-1640 medium supplemented with 5% human AB serum. Cells were then seeded into 96-well plates (1 × 10^6^ cells/well), incubated for 2 h adding (or not) EVs in the culture (200 × 10^6^/well), and stimulated with the bacterial superantigen staphylococcal enterotoxin B (SEB; 1 μg/mL) for 6 days. After incubation, cells were stained with the following mAbs: anti-CD3 (PC7), anti-CD4 (APC), and anti-CD8 (PE). Cells were incubated for 20 min at room temperature, washed, and analyzed using a Gallios flow cytometer. Results are expressed as the percentage of proliferating CD3⁺CD4⁺ and of CD3⁺CD8⁺ T cells, as determined by CFSE dilution. The inhibition of T-cell proliferation was calculated as follows: [(percentage of proliferating T cells without EVs - percentage of proliferating T cells with EVs)/percentage of proliferating T cells without EVs] × 100.

### Statistical analysis

Statistical analyses were carried out using GraphPad Prism version 5.03. Data normality was assessed by D’Agostino-Pearson omnibus test. Accordingly, parametric (*t*-test) or non-parametric (Mann-Whitney or Wilcoxon) tests have been used to compare data. Correlations were investigated by linear regression analysis and Spearman’s rank correlation coefficient. Statistical significance was reached with *P* values < 0.05.

## RESULTS

### Characterization of EVs from MM patients and HDs

Sixteen PB plasma samples were obtained from 13 MM patients (one PB sample from 10 patients and two samples from remaining 3 MM patients), as well as from 12 HDs. Plasma samples were characterized for EV size distribution and concentration using NTA. The mean size of EVs was similar between MM patients [black circles; mean ± standard error (SE): 104.8 ± 5.6 nm] and HDs (white circles; 103.8 ± 6.9 nm) [Figure 1A]. In contrast, EVs concentration was significantly higher in plasma samples from MM patients [mean ± standard deviation (SD): 4.1 ± 0.56 × 10^11^ particles/mL] than in those from HDs (2.0 ± 0.38 × 10^11^ particles/mL; *P* = 0.0072) [Figure 1B].

**Figure 1:**
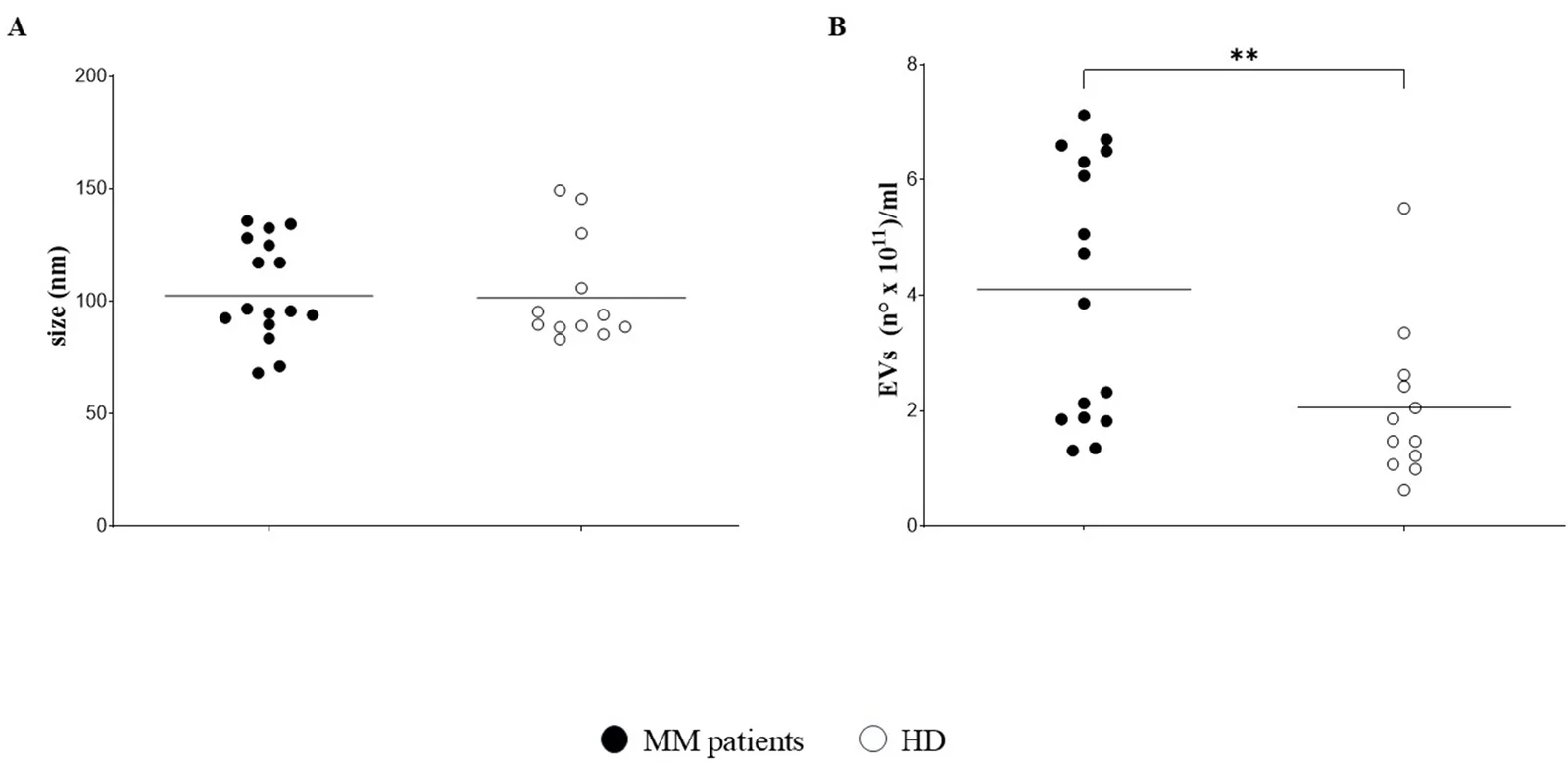
Characterization of EVs. EVs were characterized in 16 PB plasma samples from 13 MM patients and 12 HDs by NTA. Size distribution (nm) is shown in (A), whereas concentration (particles × 10^11^/mL) is shown in (B). Horizontal bars indicate the means. Asterisk indicates statistically significant differences as calculated by the Mann-Whitney test (^**^P ≤ 0.01). EVs: Extracellular vesicles; PB: peripheral blood; MM: multiple myeloma; HDs: healthy donors; NTA: nanoparticle tracking analysis.

### Expression of CD138, PBEF, CD38 and CD73 is increased in MM patients’ EVs

We next analyzed EVs for surface expression of the tumor-associated marker CD138, as well as NAMPT/PBEF1 and adenosinergic molecules CD38, CD39, CD203a, and CD73. The expression level of CD138, measured as MRFI, was higher in EVs isolated from MM patients (*n* = 10; MRFI mean ± SE: 7 ± 2.18) than in those from HDs (*n* = 12; 2.53 ± 0.47; *P* = 0.04) [Figure 2A]. Similarly, the expression of PBEF1, CD38, and CD73 was significantly higher in EVs from MM patients (14.5 ± 4.8, 484.2 ± 256, and 7.43 ± 2.33, respectively) than in those from HDs (4.7 ± 0.67, *P* = 0.04; 79 ± 28.8, *P* = 0.05; and 2.36 ± 0.29, *P* = 0.01, respectively). In contrast, the expression of CD203a and CD39 was comparable in EVs from MM patients (53.2 ± 28.8 and 36.8 ± 12.3, respectively) and HDs (19.0 ± 15 and 16.5 ± 3.3, respectively). A representative staining is shown in Figure 2B.

**Figure 2:**
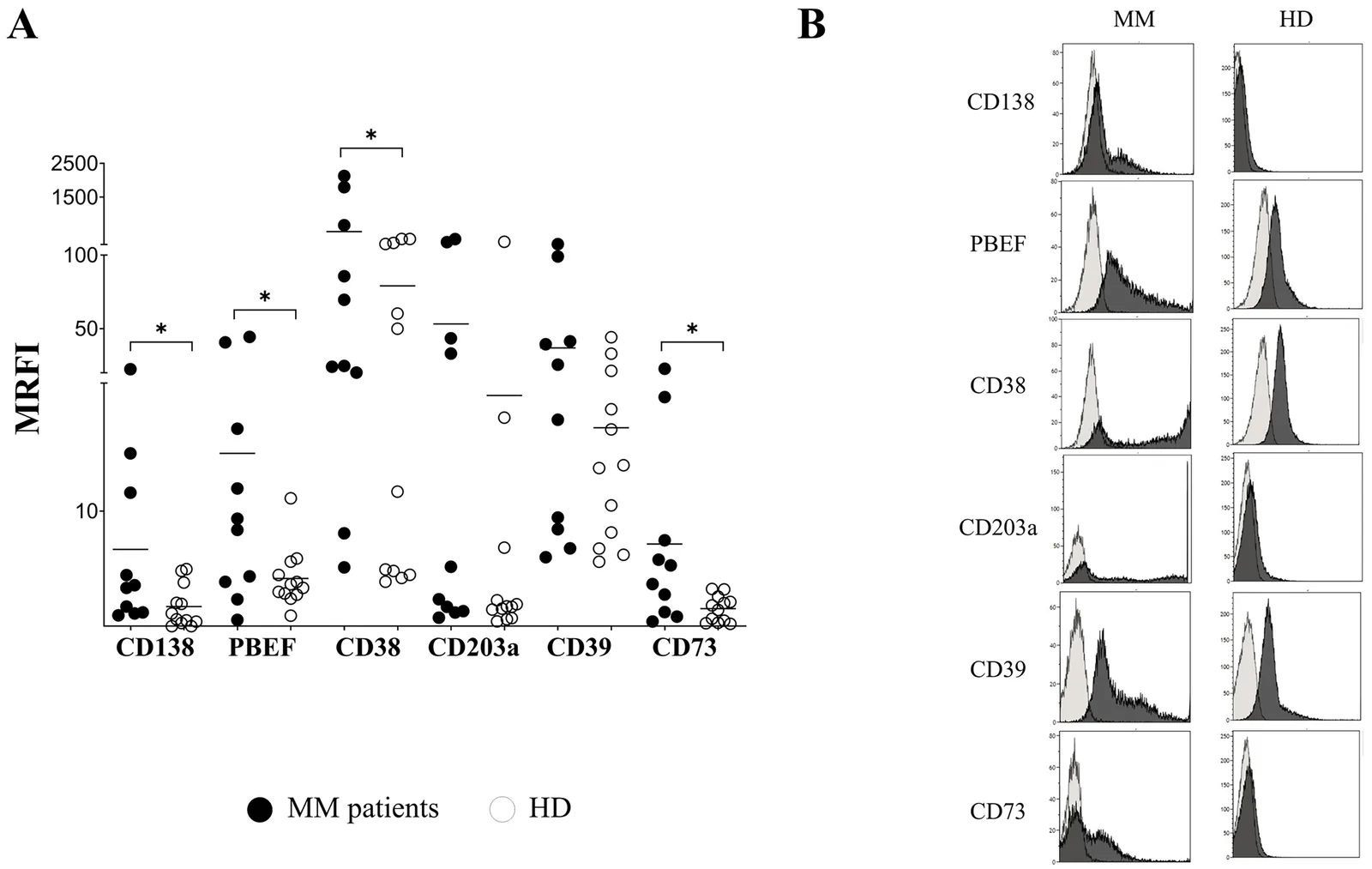
Immunophenotype of EVs. (A) EVs from PB plasma samples obtained from MM patients (*n* = 10, black dots) and HDs (*n* = 12, white dots) were analyzed by flow cytometry to assess the expression of CD138, NAMPT/PBEF1 (PBEF), CD38, CD203a, CD39, and CD73. Data are expressed as MRFI. Horizontal bars indicate the mean. Asterisk indicated statistically significant differences calculated using the Mann-Whitney test or unpaired t test, depending on data distribution (^*^P ≤ 0.05). A representative experiment is shown in (B). Dark grey profiles indicate staining with specific mAbs, whereas light grey profiles indicate staining with irrelevant isotype-matched mAbs. EVs: Extracellular vesicles; PB: peripheral blood; MM: multiple myeloma; HDs: healthy donors; NAMPT/PBEF1 (PBEF): nicotinamide phosphoribosyltransferase/pre-B-cell colony-enhancing factor 1; MRFI: mean relative fluorescence intensity; mAbs: monoclonal antibodies.

Next, we investigated whether phenotypic differences could be observed in EVs derived from malignant PC (identified as CD138^+^) and those released by healthy cells (i.e., CD138^-^) in the PB of MM patients. To address this issue, we compared the expression of NAMPT/PBEF1 and adenosinergic ectoenzymes in CD138^+^ and CD138^-^ EVs across 22 EV preparations from plasma samples of10 MM patients. As shown in Figure 3A, the expression of NAMPT/PBEF1, CD38, CD39, and CD73 was significantly higher in CD138^+^ (6.2 ± 1.4, 4.89 ± 0.9, 4.0 ± 4.3, and 2.64 ± 0.21, respectively) than in CD138^-^ EVs (1.47 ± 0.24, *P* < 0.0001; 2.63 ± 0.45, *P* < 0.001; 1.81 ± 0.39, *P* = 0.0007; and 1.3 ± 1.34, *P* = 0.03, respectively). In contrast, the expression of CD203a did not differ significantly between CD138^+^ (2.48 ± 0.22) and CD138^-^ EVs (1.47 ± 0.39). Taken together, these data suggest that EVs released from malignant MM cells are enriched in molecules with immune-inhibitory potential, an aspect examined in the subsequent section. A representative staining is shown in Figure 3B.

**Figure 3:**
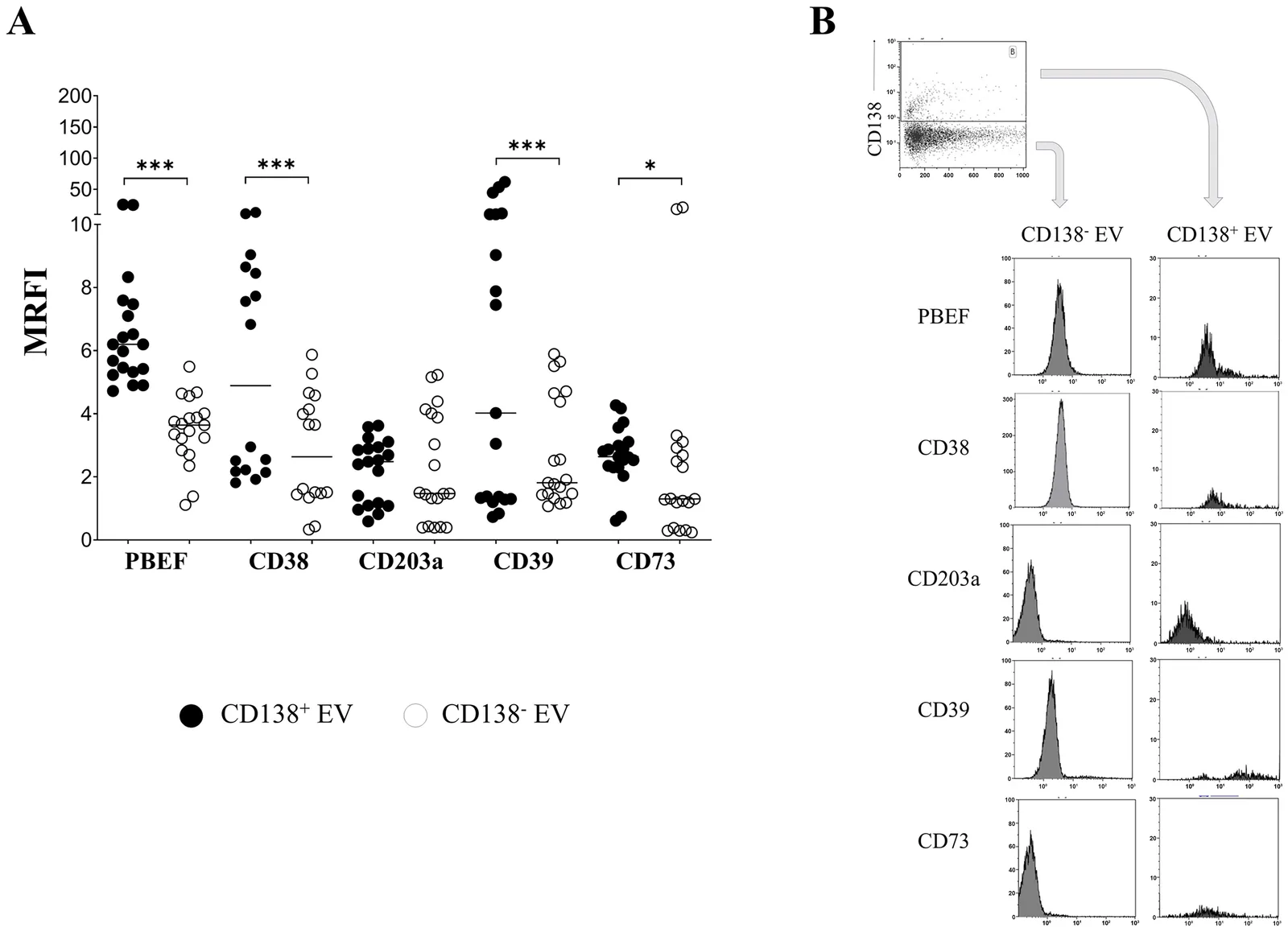
Immunophenotype of CD138^+^ and CD138^-^ EVs. (A) EVs from PB plasma samples (*n* = 22) obtained from 13 MM patients were analyzed by flow cytometry to assess the expression of NAMPT/PBEF1 (PBEF), CD38, CD203a, CD39, and CD73 in CD138^+^ (black dots) and CD138^-^ (white dots) EVs. Data are expressed as MRFI. Horizontal bars indicate the mean. The asterisk indicates statistically significant differences calculated using the Wilcoxon matched-pairs signed-rank test or paired t test, depending on data distribution (^*^P ≤ 0.05, ^***^P ≤ 0.001); (B) shows a representative experiment. Dark grey profiles indicate staining with specific mAbs, whereas light grey profiles correspond to staining with irrelevant isotype-matched mAbs. A plot showing CD138 expression on EVs and the gating strategy is shown in the upper panel. EVs: Extracellular vesicles; PB: peripheral blood; MM: multiple myeloma; NAMPT/PBEF1 (PBEF): nicotinamide phosphoribosyltransferase/pre-B-cell colony-enhancing factor 1; MRFI: mean relative fluorescence intensity; mAbs: monoclonal antibodies; CD: cluster of differentiation.

### Immune responses are modulated by MM patients’ EVs *in vitro*

We next tested the ability of EVs obtained from MM patients’ PB plasma samples to regulate immune effector cell functions *in vitro*. To address this issue, MNCs from HD were stimulated with SEB in the presence or absence of MM-derived EVs. As shown in Figure 4A, CD4⁺ and CD8^+^ T-cell proliferation after stimulation with SEB (% proliferating cells; mean ± SD: 86.5 ± 4.0 and 79.3 ± 10.8, respectively) was downregulated when MM-derived EVs were added (61.2 ± 16.3, *P* = 0.01 and 54.8 ± 15.5, *P* = 0.01, respectively).

**Figure 4:**
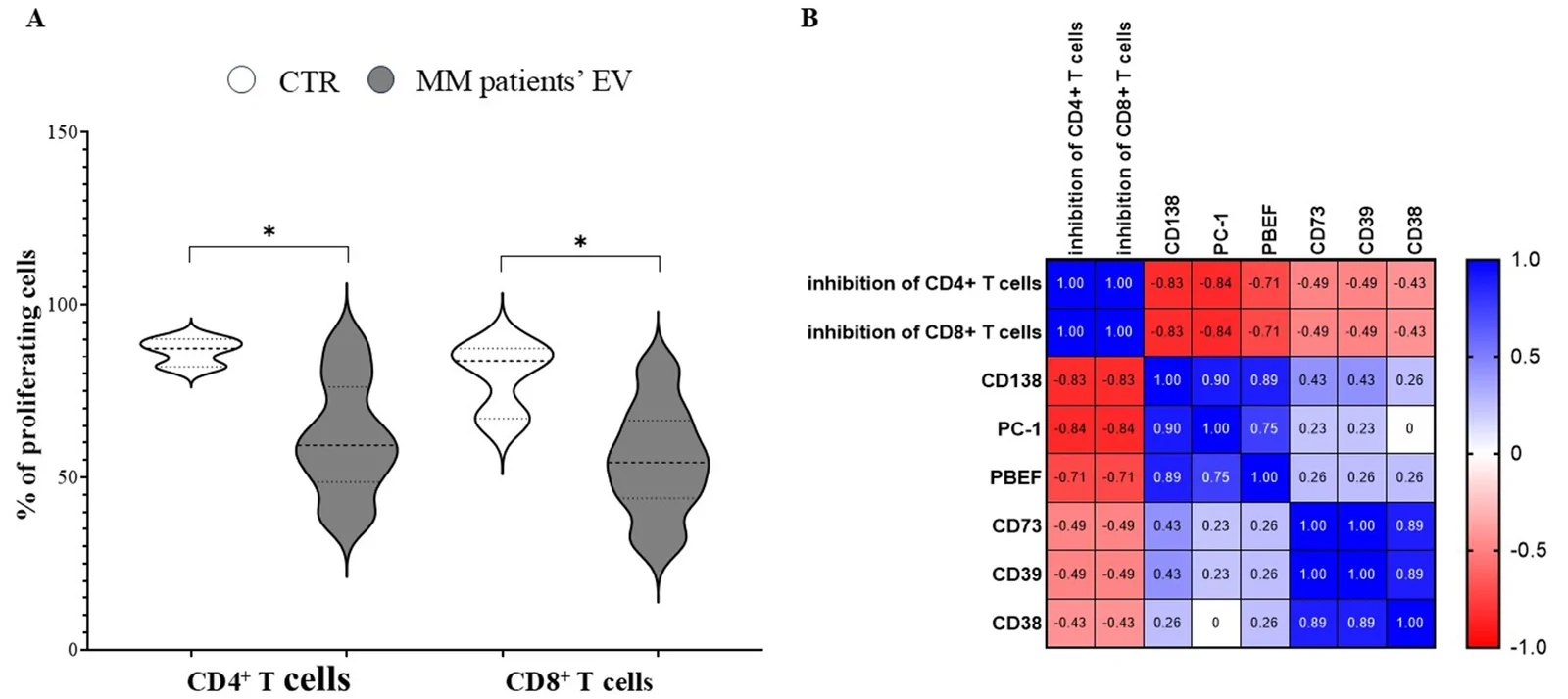
Immune-regulatory properties of MM-derived EVs. (A) Violin plots indicate the percentage of CD4^+^ and CD8^+^ T cells that proliferated after SEB stimulation, in the presence (grey profiles) or absence (white profiles) of MM-derived EVs. Profile spikes indicate maximum and minimum values, whereas dotted horizontal lines indicate quartiles and medians. Statistically significant differences were calculated using the Mann-Whitney test (^*^P ≤ 0.05) and are indicated by an asterisk. Correlations between different parameters, calculated by linear regression, are shown in the correlogram in (B) including (i) inhibition of CD4^+^ or CD8^+^ T cell proliferation and (ii) expression of different molecules on EVs. Blue indicates positive correlations (between 0 and 1), whereas red indicates inverse correlations (between 0 and -1). CTR: Control; MM: multiple myeloma; EVs: extracellular vesicles; SEB: staphylococcal enterotoxin B; PBEF: pre-B-cell colony-enhancing factor; PC-1: plasma cell antigen-1 (ectonucleotide pyrophosphatase/phosphodiesterase 1, ENPP1); CD: cluster of differentiation.

Finally, we assessed potential correlation between the down-modulation of T-cell proliferation and expression of surface markers on MM patients’ EVs (i.e., CD138, NAMPT/PBEF1, CD38, CD39, CD73, and CD203a). As shown in the correlogram in Figure 4B, inhibition of CD4⁺ and CD8^+^ T-lymphocyte proliferation correlated with the expression of CD138, CD203a, and NAMPT/PBEF1 (PBEF), and, to a lesser extent, with CD38, CD39, and CD73 on MM-derived EVs. However, statistically significant correlations were observed only between CD4⁺ and CD8^+^ T-cell proliferation and the expression of CD138 (*P* = 0.029) and CD203a (*P* = 0.0022) on EVs.

## DISCUSSION

In recent years, several studies have acknowledged the involvement of EVs in tumor progression and tumorigenesis both in solid and hematological malignancies^[26,27]^. In addition, EVs are used as biomarkers for diagnostic purposes and for monitoring the course of different tumors. They have also been proposed as potential targets for novel therapeutic strategies^[28,29]^. Indeed, tumor-derived EVs can modulate the tumor microenvironment by mediating intercellular communication, thereby promoting immune escape, metastatic dissemination and resistance to therapy^[24,27]^. Importantly, EVs may provide insights into disease states and serve as diagnostic tools for early cancer detection, as they reflect gene mutations as well as phenotypic and functional features of their cells of origin, enabling minimally invasive tracking of disease progression^[30-32]^.

For this reason, the characterization of circulating tumor-derived EVs in the PB as a form of liquid biopsy has attracted considerable interest for diagnostic and therapeutic purposes in recent years^[30,33-35]^. Accordingly, several studies have examined EVs in the PB of patients with solid and hematological malignancies, characterizing their role in tumor progression as well as their diagnostic and prognostic value^[33,36-41]^. More recently, Reale and coworkers described different methods for EVs isolation and characterization from the PB of patients with MM, highlighting their relevance for diagnostic and monitoring purposes^[42]^. However, detailed phenotypic and functional characterization of these EVs remains limited. The present study addresses this gap.

Here we report that PB samples from MM patients, compared with those from HD, contained an increased concentration of EVs, while displaying a similar size distribution. Increased levels of circulating EVs compared with healthy subjects have already been reported in patients with lung cancer, where they correlate with disease stage^[43]^. We also found that EVs obtained from MM patients’ PB samples displayed higher expression levels of CD138, NAMPT/PBEF1, CD38, and CD73 than those from HDs. The increased expression of CD138 on EVs from MM patients confirms that tumor-derived, CD138-expressing EVs are detectable in the PB of MM patients, and not only in the BM niche, as previously reported^[21]^. Nonetheless, the detection of CD138 on EVs from HDs, although at lower levels, was not entirely unexpected. Indeed, CD138 can be detected on normal epithelial cells, normal PCs, and other healthy tissues^[44,45]^, and its expression can be increased during inflammation^[46]^ or in autoimmune and inflammatory diseases^[47,48]^.

The finding that NAMPT/PBEF1 is expressed at higher levels in EVs from MM patients than in those from HDs is novel and of particular interest. NAMPT/PBEF1 is a rate-limiting enzyme involved in NAD^+^ biosynthesis, and its expression has been reported in MM, where is crucial for tumor cell growth and osteoclast activity^[18]^. More importantly, NAMPT/PBEF1 expression has been shown to correlate with disease progression^[49]^. In addition, it has been proposed both as a therapeutic target^[50]^ and as a biomarker of chemotherapy efficacy in patients with NDMM, particularly in combination with nicotinate phosphoribosyltransferase (NAPRT)^[51]^. Among the adenosinergic pathways, we observed significantly higher expression of CD38, which belongs to the non-canonical pathway, and of CD73, which represents a functional bridge between the canonical and non-canonical pathways. These data are consistent with our previous observations on EVs obtained from BM samples of MM patients^[21]^. Accordingly, EVs potentially capable of producing the immunosuppressive molecule ADO from different substrates, including ATP and NAD^+^, are detectable in the PB of MM patients and not only within the BM microenvironment. Furthermore, we demonstrated that CD138^+^ EVs, which are mostly derived from malignant PCs (although the contribution of CD138^+^ healthy cells cannot be excluded), expressed significantly higher levels of NAMPT/PBEF1, CD38, CD39, and CD73. These data suggested that those EVs display an increased capacity for NAD^+^ and ATP metabolism leading to ADO production, as compared with EVs derived from healthy cells. Again, this feature mirrors what has been observed in EVs isolated from BM samples of MM patients^[21]^. During MM progression, the BM niche becomes enriched in extracellular nucleotides (e.g., ATP and NAD⁺), as well as dinucleotides such as cyclic Guanosine monophosphate (GMP)-AMP (cGAMP). It has been reported that both mono- and di-nucleotides may contribute to the establishment of immune tolerance^[52]^, through their metabolization by ectoenzymes expressed by BM resident cells, such as CD38, CD203a, and CD73. The resulting enzymatic reactions trigger intracellular calcium flux signaling and drive the production of extracellular ADO, thereby linking purinergic metabolism to immunosuppressive processes^[4]^.

In this scenario, the interplay between the DNA damage response and purinergic metabolism remodeling is increasingly recognized as a critical determinant of MM adaptation to therapy. Genotoxic stress induces the release of ATP, NAD⁺, and cGAMP, which are rapidly processed by ectonucleotidases in the MM microenvironment. Among these ectonucelotidases, CD203a acts as a central regulator by converting ATP and NAD⁺ to AMP^[53]^. Thus, CD203a activity fuels the non-canonical adenosinergic pathway independently of the enzymatic function of CD39^[9]^. Furthermore, CD203a can suppress cGAS-Stimulator of Interferon Genes (STING)-mediated innate immune activation by degrading extracellular cGAMP^[53]^. In MM, PCs and BM stromal cells frequently upregulate CD203a, enabling efficient conversion of therapy-induced nucleotides into AMP and sustaining CD73 dependent ADO accumulation^[54]^. The combined enhancement of ADO signaling and the attenuation of STING responses promotes an immunosuppressive microenvironment that supports MM cell survival and contributes to therapeutic resistance.

Data from the functional studies reported here indicate that EVs obtained from MM patients’ PB samples may exert immune-suppressive activities, as evidenced by their ability to dampen T-cell proliferation *in vitro* in both helper and cytotoxic T lymphocytes. Previous studies on EVs derived from patients with MM have reported inhibitory effects on osteogenesis and angiogenesis *in vitro*^[55,56]^.

Several studies support the concept that EVs in MM act as key mediators that reprogram the BM microenvironment into an immunosuppressive niche, thus facilitating tumor progression through multiple, parallel mechanisms. One of the primary mechanisms involves the polarization of macrophages toward a pro-tumorigenic M2 phenotype. Exosomal miR-let-7c derived from mesenchymal stem cells drives this M2 shift, while MM-derived exosomes specifically increase the expression of M2-associated markers such as Arginase (Arg)-1, interleukin (IL)-10, and CD206 in resting macrophages^[57,58]^. Furthermore, tumor-derived small EVs induce the expression of Programmed Death-Ligand (PD-L)-1 and pro-inflammatory cytokines such as IL-6 in macrophages by activating the Signal Transducer and Activator of Transcription (STAT) 3 and Toll-like receptor (TLR) 4/Nuclear factor kappa-light-chain-enhancer of activated B cells (NF-κB) signaling pathways^[59]^. Beyond macrophages, EVs are instrumental in the expansion and activation of myeloid-derived suppressor cells (MDSCs). In this context, Bone Marrow Stromal Cells (BMSC)-derived exosomes are internalized by MDSCs, directly promoting their survival through activation of STAT1 and STAT3 signaling and upregulation of the anti-apoptotic proteins B-cell lymphoma-extra large (Bcl-xL) and Myeloid Cell Leukemia-1(Mcl-1)^[60]^. T-cell activity is likewise severely compromised through multiple EV-mediated pathways. Hagiwara *et al*. reported that EVs derived from MM promote T-cell exhaustion by upregulating immune checkpoint markers such as PD-1, T-cell immunoreceptor with Ig and ITIM domains (TIGIT), and Lymphocyte-activation gene (LAG) 3 on CD8^+^ T cells, a process largely driven by Sphingosine kinase (SPHK) 1/Sphingosine-1-phosphate (S1P) signaling^[61]^. Moreover, conditioning of the BM microenvironment with these vesicles increases PD-1 expression specifically on CD4^+^ T cells^[62]^.

Here, we also report that circulating EVs in PB are equipped with complete adenosinergic pathways (CD38, CD39, CD203a, and CD73). This feature enables them to produce the immunosuppressive molecule ADO. Thus, circulating EVs are capable of dampening both CD4^+^ and CD8^+^ T-cell proliferation at sites far from the primary tumor. Natural killer (NK) cells are also targeted by EV-mediated mechanisms, since chronic exposure to EVs carrying the MHC class I polypeptide-related sequence A (MICA) ligand leads to downmodulation and degradation of the Natural Killer Group (NKG) 2D receptor and can even trigger “fratricide” when MICA is transferred directly from vesicles to the NK-cell surface^[63]^. In addition, exosomal lncRNA Nuclear Paraspeckle Assembly Transcript (NEAT) 1 inhibits NK-cell activity and cytokine production by downregulating Pre-B-cell leukemia transcription factor (PBX) 1 through an Enhancer of zeste homolog (EZH) 2-mediated axis^[49]^. Finally, MM EVs are enriched in Complement Component 4 Binding Protein Alpha (C4BPA), which helps tumor cells evade the host complement system^[64]^, and phosphatidylserine-positive vesicles contribute to a highly immunosuppressive state^[65]^.

We have further recently demonstrated that EVs obtained from BM samples of MM patients regulate multiple immune cell functions, including T-cell proliferation and cytokine release. Those effects are partly mediated by the immune checkpoints Human Leukocyte Antigen (HLA)-G and PD-L1 expressed on their surface^[66]^. Moreover, EVs from BM samples of MM patients express functional adenosinergic ectoenzymes, paralleled by the production of ADO from ATP and NAD^+^^[21]^. Based on these observations, we previously hypothesized that EVs released within the BM microenvironment may modulate local anti-tumor immune responses. Furthermore, those EVs may arrive in the PB and, from there, they reach other BM niches or other organs (see Graphical Abstract). Here we provide the first evidence that EVs detected in MM patients’ PB samples have potential immunomodulatory effects. It is conceivable that these EVs may recirculate through the blood stream to perform these modulatory activities on immune cells at distant sites, rather than in the PB. Downregulation of T-cell proliferation *in vitro* positively and significantly correlated with the expression of CD138 and CD203a/PC-1 on EVs, suggesting that these effects can be attributed, at least in part, to the presence of tumor-derived EVs expressing a functional non-canonical adenosinergic pathway. Nonetheless, these findings require validation using specific ectoenzyme inhibitors, and we cannot exclude the contribution of additional immune-suppressive mechanisms. Further studies will therefore be necessary to validate the data here presented. Nonetheless, we provide the first evidence that immune-regulatory EVs derived from MM cells are clearly present in the PB of MM patients. Those EVs may be involved in tumor-derived immune-suppression in the periphery and, presumably, in tumor progression.

This study has limitations, including the relatively small number of MM patients analyzed. Although the use of primary patient samples inevitably introduces a degree of clinical heterogeneity, the application of strict inclusion criteria, uniform pre-analytical processing, and standardized functional assays mitigates this limitation. Nevertheless, future studies based on larger and clinically stratified cohorts will be essential to dissect the relative contribution of disease stage, comorbidities, and prior treatments to EV-mediated immune modulation in MM. Another limitation is the lack of experiments employing specific inhibitors of adenosinergic ectoenzymes to directly assess their role in the immune-regulatory properties of MM-derived EVs. Despite these limitations, our findings, together with results from other groups, underscore the relevance of EVs found in the PB of MM patients. Circulating CD138^+^ microparticle levels accurately reflect tumor burden, often signaling early relapse weeks before symptoms or standard tests show any change^[65]^. The proteomic content of EVs, specifically the enrichment of CD44, is a key indicator of corticosteroid resistance and a predictor of shorter overall survival^[67]^. Beyond tracking the tumor itself, MM-EVs inhibit bone formation through transfer of molecules like miR-103a-3p, providing a precise biomarker for the diagnosis of bone lesions^[68]^. EVs may also enable real-time monitoring of treatment failure, as the presence of P-glycoprotein and phosphatidylserine on microparticles correlates with multidrug resistance and aggressive disease progression^[65]^. Finally, it has been established that a high EV cargo ratio (> 0.6 × 10^8^ particles) is a significant prognostic parameter associated with immune dysfunction and lower survival rates, supporting a more personalized approach to long-term clinical management^[64]^. In conclusion, PB EVs may represent a valuable tool for the diagnosis and monitoring of MM, potentially overcoming some limitations of invasive BM procedures; however, additional studies are required to confirm these hypotheses.

## DECLARATIONS

### Availability of data and materials

Raw data are available upon reasonable request from the corresponding author.

### Copyright

© The Author(s) 2026.
